# Functions and Mechanisms of Fibroblast Growth Factor (FGF) Signalling in *Drosophila melanogaster*

**DOI:** 10.3390/ijms14035920

**Published:** 2013-03-14

**Authors:** Villö Muha, Hans-Arno J. Müller

**Affiliations:** Division of Cell and Developmental Biology, College of Life Sciences, University of Dundee, Dundee DD15EH, Scotland, UK; E-Mail: v.muha@dundee.ac.uk

**Keywords:** fibroblast growth factor, development, cell signalling, cell migration, differentiation, *Drosophila*

## Abstract

Intercellular signalling via growth factors plays an important role in controlling cell differentiation and cell movements during the development of multicellular animals. Fibroblast Growth Factor (FGF) signalling induces changes in cellular behaviour allowing cells in the embryo to move, to survive, to divide or to differentiate. Several examples argue that FGF signalling is used in multi-step morphogenetic processes to achieve and maintain a transitional state of the cells required for the control of cell fate. In the genetic model *Drosophila melanogaster*, FGF signalling via the receptor tyrosine kinases Heartless (Htl) and Breathless (Btl) is particularly well studied. These FGF receptors affect gene expression, cell shape and cell–cell interactions during mesoderm layer formation, caudal visceral muscle (CVM) formation, tracheal morphogenesis and glia differentiation. Here, we will address the current knowledge of the biological functions of FGF signalling in the fly on the tissue, at a cellular and molecular level.

## 1. *Drosophila melanogaster* as a Versatile Model for FGF Signalling Research

Fibroblast Growth Factors (FGFs) were first discovered in mammals and the horizon of insect FGF research opened up with the discovery of the first FGF receptor (FGFR) gene in *Drosophila melanogaster*, suggesting that FGF signalling is evolutionary conserved [1]. *Drosophila* is an attractive model to study FGF signalling, because its low genetic redundancy facilitates functional studies. The *Drosophila* genome encodes only two FGFRs [Heartless (Htl) and Breathless (Btl)] and three FGF ligands (Pyramus (Pyr), Thisbe (Ths) and Branchless (Bnl)) that can combine into three functional interactions of FGFR/FGF-ligand pairs: Htl/Pyr, Htl/Ths and Btl/Bnl. In contrast, in humans presumably more than 70 FGFR/FGF combinations are generated by four FGFRs and 22 FGF ligands, respectively; alternative mRNA splicing increases the FGFR repertoire from four to seven proteins [2,3]. Moreover, from the possible FGFR/FGF combinations, human cells may utilize more than one at the same time and these sometimes trigger different, even antagonistic intracellular signals. In *Drosophila*, the *htl* and *btl* genes are expressed in distinct tissues and during different developmental times, thus providing independent models to investigate FGF signalling pathways in many different developmental processes.

Over 100 years of *Drosophila* research generated a wide range of genetic tools helping to understand the biological function of gene networks in a developmental context. Both, forward and reverse genetic approaches have been extensively applied in *Drosophila* FGF signalling studies. For example, aberrant migration of tracheal cells in embryos deficient for the *btl* locus implied a function of FGF signalling in cell motility [1,4] and Pyr and Ths were identified in screens for genes involved in mesoderm development [5–7]. Furthermore components of FGF signalling pathways have been identified in genetic screens and their relationship to FGFR activation has been resolved. A broad range of mutant alleles and transgenic constructs including dominant negative (DN) and constitutively active (CA) FGFR constructs [8,9] have been employed to determine tissue specific signalling events and epistatic relationships [10,11].

The two *Drosophila* FGFRs are implicated in similar cellular contexts as in vertebrates such as proliferation, cell survival, differentiation and cell migration and in some instances FGF signalling is even involved in similar developmental processes in flies and mammals. The formation of intricate branching patterns of the respiratory system, lungs and trachea, is controlled by similar ontogenetic principles in their formation, although they are evolutionary convergent structures. Branch formation of both organs is orchestrated by FGF signalling: Fgfr2-IIIb/FGF10 in human and Btl/Bnl in *Drosophila*[12]. During gastrulation in vertebrates and invertebrates FGF signalling plays a crucial role in directing cell migration. In the early mouse and chick gastrula, FGF4 and FGF8 direct the migration of epiblast cells out of the primitive streak [13,14]. Similarly FGF8-like ligands, Pyr and Ths, serve as a guidance cue for Htl expressing mesoderm cells to spread along the ectoderm [15–17]. Thus, FGF provides conserved signalling mechanisms employed for the formation of the germ layers in gastrulation and the specification of mesodermal, endodermal and ectodermal derivatives in insects and vertebrates.

## 2. Biological Functions of FGF Signalling in *Drosophila* Development

A limited number of signalling pathways are applied repetitively and in combination to control growth, patterning and differentiation throughout development and to maintain normal cellular functions in multicellular organisms. In *Drosophila*, FGF signalling has been investigated exclusively during development and little is known about its function in organ and tissue homeostasis in adult flies. FGFs exhibit diverse roles and participate in the morphogenesis of several organs with distinct origins. Most has been learned from studies on mesoderm morphogenesis and tracheal development controlled by Htl and Btl respectively. Htl is also indispensable for gliogenesis and nervous system development. In all cases the biological processes are complex, multi-step events where FGF signalling is reiteratively used.

### 2.1. Biological Functions of Htl

**Figure 1:**
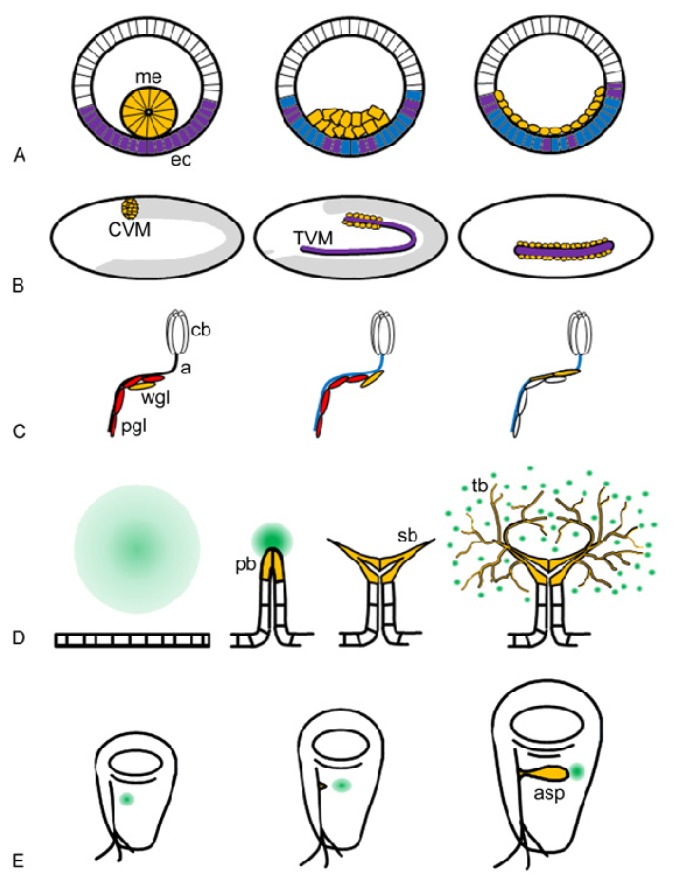
Developmental functions of FGF signalling in *Drosophila*. Diagram of morphogenetic events regulated by FGF signalling. FGFR (Htl or Btl) expressing cells are coloured orange, while expression of FGF ligands is indicated with separate colours: Bnl green, Pyr red, Ths blue, Pyr+Ths purple. (**A**–**C**) show Htl-dependent and (**D**–**E**) Btl-dependent multi-step developmental processes. (**A**) Schematic representation of cross sections of embryos during mesoderm layer formation illustrating tube stage, flattening and layer formation (me—mesoderm, ec—ectoderm). (**B**) Caudal visceral mesoderm (CVM) cell migration is drawn as longitudinal sections of the embryo. Posterior mesoderm cells migrate along the trunk visceral mesoderm (TVM) to form the progenitors of the longitudinal gut muscle. (**C**) Glial development in the eye imaginal disc: Glia cells migrate along the perineural glia sheet, once glia-axon contact is formed, glial wrapping commences (pgl—perineurial glia, wgl—wrapping glia, cb—cell body, a—axon). (**D**) Diagram of trachea development showing primary branch budding, secondary branch formation and ramification of terminal cells according to the oxygen need of the supplied cell (pb—primary branch, sb—secondary branch, tb—terminal branches). (**E**) Dorsal air sac forms from a tracheal branch that is associated with the wing imaginal disc (asp—air sac primordium).

Htl signalling is required for multiple successive cell behaviours during mesoderm layer formation and differentiation [8,18]. During gastrulation, Htl is essential for the establishment of the mesoderm layer that subsequently differentiates into specific mesodermal lineages such as heart, visceral mesoderm, somatic muscles and fat body. Mesoderm layer formation can be described as a sequence of successive steps: tube stage, tube collapse, flattening and spreading (Figure 1A) [19]. Mesoderm cells express Htl, while the underlying neuroectoderm cells express Pyr and Ths [5,7]. The mesoderm originates from invaginated epithelial cells, which initially form a tube-like structure. Invagination is independent of Htl, but collapse of the tube depends on FGF signalling [6,20]. FGF signalling is important for the formation of cell–cell contact between ectoderm and mesoderm cells resulting in symmetrical positioning of cells into a multi-layered aggregate onto the underlying ectoderm [21]. Following tube collapse, mesoderm cells move radially towards the ectoderm, described as flattening behaviour [16]. Upon flattening, cells at the edge of the aggregate switch from radial to dorsal movement and this initiates spreading of the cell collective in dorsal direction [21,22]. In addition to dorsal migration, cells in the interior of the aggregate intercalate radially. The driving force for radial intercalation is unclear, but it requires both Ths and Pyr activity [16]. Cell rounding during mitosis has been suggested to contribute to intercalation [21,22], but mitosis is not essential because *string* (*stg*) mutant embryos where no post-blastoderm mitoses occur produce a normal mesoderm layer [23]. Initially the expression patterns of *pyr* and *ths* are overlapping thus supporting robust signalling during collapse and flattening. During dorsal migration Ths remains expressed in the ventrolateral neuroectoderm and plays a role in radial movement while Pyr is now expressed in the dorsal ectoderm serving as a guiding attractant for dorsal migration and contributing to radial intercalation movements (Figure 1A) [7,15,24].

Mesoderm layer formation is a pivotal morphogenetic event, because it provides the cells with defined positional information along the dorsal-ventral axis. Cells in the mesoderm layer receive patterning cues from the underlying ectoderm, which eventually drives them into distinct differentiation programs. With exception of the fat body, all other mesoderm derivatives require Htl input. Cardiac mesoderm is originated from the dorsal most two rows of cells of the mesoderm layer; in *htl* null-mutants the mesoderm fails to reach this dorsal position causing lack of heart cell differentiation [25]. Htl signalling is important for muscle cell fate by maintaining transcription of Myocyte enhancing factor 2 (Mef 2), a key regulator of somatic muscle differentiation [26]. The ventral-most portion of the mesoderm layer gives rise to somatic muscles, which are reduced and abnormally arranged in *htl* mutant embryos. The adult somatic muscles are built up from multi-nucleated myofibres arisen from fusion of founder cells with myoblasts. In the pupae Htl signalling regulates adult founder cell formation [27]. Htl is also involved in the morphogenesis of visceral mesoderm derivatives by directing the migration of caudal visceral mesoderm cells (CVM) (Figure 1B) [8]. The caudal group of visceral mesoderm cells are the founder cells of the longitudinal gut muscle myoblasts. They express Htl and they actively migrate along the *pyr* and *ths* expressing trunk visceral mesoderm (TVM) towards the anterior of the embryo [28,29]. Pyr and Ths act redundantly to provide directionality and without FGF ligands, CVM cells go astray, move slower, detach from the TVM and eventually die [28,29].

Apart from its central role in mesoderm development, Htl is also essential in the morphogenesis of neuroectoderm-derived glia in the nervous system. FGF signalling promotes elongation and migration of glial cells around the axons, an important process that precedes enveloping axonal processes and thus providing insulation during neuronal activity (Figure 1C). The embryonic central nervous system runs in two rows of longitudinal axon tracts on each side of the ventral midline surrounded by their longitudinal glia partners that originated from the lateral edge of the embryonic CNS. FGF signalling from Htl directs longitudinal glia cells to enwrap longitudinal axon tracts [25]. In the peripheral nervous system of the larvae, Htl signalling provokes migration of the glia cell population and then induces unsheathing of ommatidial axon fascicles in the eye imaginal disc [30]. The functions of Pyr and Ths are clearly separated with Pyr being responsible for glia cell migration and Ths being required for differentiation. In the larval CNS, Htl controls gliogenesis in both perineural and cortex glia cells [31].

### 2.2. Biological Functions of Btl

The developmental functions of Btl are distinct from those of Htl and no case has been reported where the two FGFRs act in concert to control a single morphogenetic event. Btl orchestrates successive steps of tracheal morphogenesis by inducing clearly distinct cellular events. The tracheal system is the respiratory organ of insects and is formed as a multicellular, branched tubular network. It develops by sequential sprouting of primary, secondary, and terminal branches from an epithelial sac of ~80 cells in each body segment of the embryo (Figure 1D) [32]. First, six tracheal buds form and elongate into primary tubular epithelial branches. Next, secondary branches sprout from the tip of the primary branches, surrounding the tracheal lumen by a single cell. Finally, during larval stages long cytoplasmic extensions form a fine treelike structure of terminal branches that deliver oxygen and gases to the internal tissues and in which the lumen forms within the terminal branch cells.

The specification of tracheal progenitor cells does not depend on Btl, as in *btl* mutants the number of tracheal cells and the formation of tracheal pits proceeds normally [4]. The primary phenotype of *btl* is the failure of primary branch formation: the dorsal trunk, visceral branches and lateral branches are missing suggesting that budding of tracheal precursor cells from the placode is impaired [4]. Btl activity is also required for subcellular lumen morphogenesis of secondary branches and for ramification of terminal branches [11,33,34]. Tracheal cells respond to Btl signalling with budding or branching depending on their differentiation state. Moreover, Btl itself induces the expression of sets of key secondary and terminal branch genes including *pointed* and *blistered*, thus ensuring different cellular responses [12].

Btl activity is highly regulated both spatially and temporally to sustain normal patterning of tracheal branching. The key determinant controlling Btl activity is the dynamic expression of its ligand Bnl, which is regulated both developmentally and in response to exogenous stimuli (Figure 1D). During primary branch formation mesenchymal tissue around each tracheal sac expresses *bnl*. Primary and secondary branch patterning follows a reproducible pattern of *bnl* expression in all embryos. The fine-tuning of terminal branches depends on the demand of oxygen in the tissue. Low oxygen levels stimulate the expression of Bnl and thereby promote branch initiation in terminal cells [33]. Tracheal cells can simultaneously sense two nearby sources of Bnl and respond by directional growth of their lumen and extension of long cytoplasmic protrusions in a dosage-sensitive fashion. Indeed, Bnl/Btl-dependent tracheal morphogenesis provides one of the best-known examples demonstrating the function of FGFs as chemo-attractants in tissue morphogenesis.

A special tracheal structure, the dorsal air sac, supplies oxygen to the adult flight muscles. The dorsal air sac develops during the third instar larval stage from a wing-disc associated tracheal branch (Figure 1E). Btl signalling induces migration of the air sac primordium (ASP) cells into the wing imaginal disc and initiates growth and differentiation of tip and stalk structures [35]. Similar to embryonic secondary branch morphogenesis, these events require the transcriptional target Pointed. The FGF and EGF receptor tyrosine kinases have distinct functions in this process: FGFR being responsible for migration and EGFR regulating cell division and cell survival [35]. Therefore, the ASP provides an excellent model system to dissect RTK pathways and to identify molecular components that regulate FGF-dependent migration, EGF-dependent proliferation or both. Large cells and long protrusions in the ASP enable precise detection and quantitative evaluation of cell migration in a tissue context. Genetic screens using Mosaic Analysis with a Repressible Cell Marker (MARCM) technique allow following subcellular markers in homozygously mutant cells in genetic mosaics and has identified Myosin heavy chain (Mhc) and the endosomal protein Stam to be essential for cell migration but not for proliferation. The Hrs/Stam complex modulates endosomal sorting and directs membrane proteins to degradation or recycling. In the ASP the Hrs/Stam complex acts as a positive regulator of Btl and ensures efficient level of FGF signalling [36,37]. Further analysis of ASP development by expanding MARCM based screens in a genome wide fashion has great potential to reveal novel players and regulatory components of FGF-dependent cell migration. A similar approach could be exploited in the developing male genitalia, where Btl/Bnl signalling recruits mesenchymal cells into the male genital imaginal disc [38].

Several distinct examples argue for the fact that Btl is important in many other developmental processes and in great majority these are migratory events in the oocyte, embryo and larvae. These processes include border cell migration during oogenesis [39], migration of ventral midline glia cells [4], migration of mesodermal cells into the male genital imaginal disc [38], and formation of the neuromuscular junction [40]. While these studies indicate the multiple roles of Btl signalling in the fly, they may also provide important assays and models to address critical questions towards the functions and mechanisms of FGF signalling in the future.

## 3. The FGF Signalling Pathway in *Drosophila*

**Figure 2:**
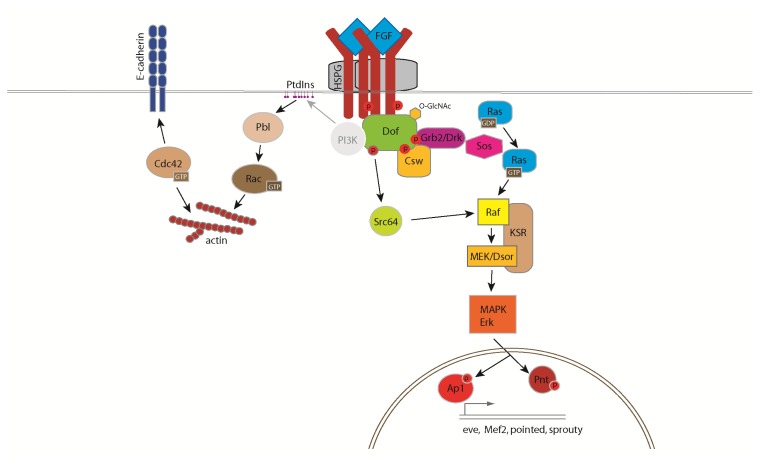
Schematic model of the FGF signalling cascade in *Drosophila melanogaster.* FGFs bind to their FGFRs and this interaction is stabilized by heparan sulfate proteoglycans (HSPG). Activation of the FGFR leads to auto- and trans-phosphorylation of their tyrosine kinase domains and to phosphorylation of its adaptor protein Dof. Dof protein is *O*-GlcNAcylated (*O*-GlcNAc) and possesses multiple clusters of tyrosine residues directing the signal towards various cascades, three of which—the Csw/Shp2, Grb2/Drk and Src64B pathways—have been proposed to contribute to MAPK activation. This route of FGF signalling is responsible for inducing gene transcription, and executing proliferative and anti-apoptotic responses. Dof also contains a putative binding site for PI3K that could locally modify the phosphatidylinositol (PtdIns) composition of the plasma membrane and thus recruit downstream signalling components for example the RhoGEF Pbl. Pbl acts on the Rac pathway to promote the formation of actin rich protrusions in the mesoderm. Actin polymerisation is required for protrusion formation in a Rac-, Cdc42- and FGF-dependent fashion. Putative interactions are indicated as grey arrows and the putative component of the *Drosophila* FGF pathway, PI3K, is represented as a circle with no border.

FGFs signal through RTKs, and thus the FGF signalling pathway shares many components with the other RTK signalling cascades. Downstream of the receptor, FGF-specific and canonical RTK signalling molecules are both responsible for signal transmission in complex cellular responses (Figure 2).

**Figure 3:**
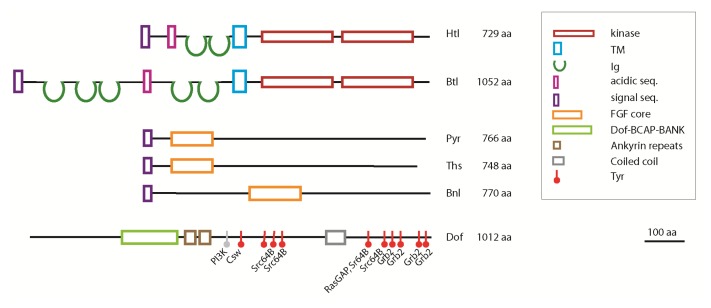
Domain structure of the *Drosophila melanogaster* FGFRs, FGFs and Dof. Htl is built up of an amino-terminal signal sequence (purple), an acidic region (pink), two Immunoglobulin-like (IG) domains (green loops), a transmembrane domain (light blue) and an intracellular split tyrosine kinase catalytic domain (red). Btl exhibits similar domain composition to Htl, but Btl contains three additional IG-like domains in the extracellular part of the protein. *Drosophila* FGF ligands possess an amino-terminal signal sequence (purple), the conserved FGF core domain (orange) and a long carboxy-terminal tail. The Dof protein contains a Dof-BCAP-BANK domain (light green) required for receptor binding, two ankyrin repeats (brown) and a coiled-coil domain (grey). Tyrosine residues that are indicated in binding of downstream signalling molecules are marked (red). The Tyrosine residue located within the consensus site for PI3K binding is coloured in light grey [49,50].

FGFs bind to their receptor and this interaction is stabilized by heparan sulfate proteoglycans (HSPG) [41,42]. HSPGs consist of a core protein to which complex heparin/heparan sulfate glycosaminoglycans are attached. Formation of a FGF:FGFR:HSPG ternary complex, where HSPG acts as co-receptor, is essential for FGF signalling. In *Drosophila* the genes encoding for HSPG biosynthesis are already expressed during oogenesis and genetic screens targeting such maternal genes have found many mutations in HSPG biosynthetic genes, classic examples of which are *sugarless* (*sgl*) encoding UDP-glucose 6-dehydrogenase and *sulfateless* (*sfl*) encoding heparan sulfate-glucosamine *N*-sulfotransferase [43,44]. Impaired biosynthesis of HSPG in *sgl* and *sfl* embryos, perturbation of the sulfation level or mutation of the protein core completely abolishes signalling as demonstrated by their mutant phenotypes resembling those of a combination of *htl* and *btl* mutants [45,46]. Different FGFRs have a preference towards specific HSPG partners: the Dally and Dally-like protein (Dlp) participate in Btl signalling but they are dispensable for Htl signalling [47], while Syndecan (Sdc) contributes to Htl-dependent mesoderm layer formation [48]. Dally, Dlp and Sdc belong to structurally distinct groups of HSPGs. Dally and Dlp are glypicans, which are attached to the membrane via GPI membrane anchor, while Sdc is a transmembrane proteoglycan. The structural requirements that reflect the specificity of HSPGs for the Htl and Btl receptors remain to be defined. The intracellular kinase domain of Htl and Btl shows high sequence homology (over 75% amino acid identity), but they differ in the their extracellular domains (Figure 3). The specificity of Btl-Bnl and Htl-Pyr/Ths receptor-ligand interactions is also reflected by structural differences in the extracellular domains of the receptors, where the number of Imunoglobin(IG)-like domains differs dramatically between Htl and Btl [24]. Domain swapping experiments support this conclusion by demonstrating that Btl cannot be activated by FGF8-like ligands and Htl cannot be activated by Bnl [17].

*Drosophila* FGFs belong to separate groups of origins: the FGF core domains of Pyr and Ths are highly homologous of the vertebrate FGF8/17/18 group [5], whereas the FGF core domain of Bnl cannot be classified into any particular chordate FGF subfamily [51]. Bnl shares a similar level of sequence similarity with many chordate FGFs and probably represents a member of a common ancestral FGF group [34,51]. The *Drosophila* FGF primary sequence contains an amino-terminal signal peptide, an FGF core domain and a long carboxy-terminal sequence with no significant homologies to other proteins [5]. Cultured cell studies demonstrated that the carboxy-terminal domains of Pyr and Ths are cleaved off suggesting that these FGFs are produced as precursors and that polypeptides containing the FGF core domain are secreted (Figure 3) [52]. The significance of this potential regulatory step is unclear as transgenes expressing carboxy-terminal truncation of Ths and Pyr are functional indicating that cleavage of the carboxy-terminal domain is not a prerequisite for secretion or receptor activation [52,53]. A case for proteolytic control of FGF ligand distribution has been made during the development of the larval ASP, where spatial restriction of FGF ligands by the extracellular matrix metallo-protease Mmp2 has been shown to concentrate FGF to the tip territory of the air sac by degrading FGF around the adjacent stalk cells [54].

Despite their different extracellular domains and separate developmental functions, the two *Drosophila* FGFRs signal through similar intracellular cascades. Ligand binding to FGFR promotes its dimerization, which results in tyrosine-phosphorylation of the receptor and of its adaptor protein Stumps/Downstream of FGF (Dof). While in vertebrates, FRS2 (FGF receptor substrate-2) provides adaptor function in *Drosophila* the FRS2-unrelated protein Dof carries out the adaptor function and binds constitutively to FGFR [55–57]. Despite the difference in their primary sequence, FRS2 and Dof share important functional characteristics by providing a scaffold for recruitment of signalling components including Grb2 and PhosphatidylinositoI-3-kinase (PI3K) (Figure 2). Lack of *Dof* function manifests in the same developmental defects as *htl* and *btl* null-mutations, indicating that Dof is essential for FGF signalling [10,49,58].

In mammalian cells, ligand binding of FGFR activates Ras/Raf-Mek-MAPK, PI3K and PLCγ-Ca^2+^ signalling pathways [59]. In the fruit fly embryo the main emphasis was placed on activation of mitogen activated protein kinase (MAPK), mostly because of the generation of an elegant detection method using an antibody specific to double-phosphorylated ERK [60]. Compared to the detailed description in mammalian systems, little is known about PI3K and PLCγ-Ca^2+^ pathways in FGF signalling in *Drosophila*. One reason for this lack of understanding in the fly is the pleiotropy of mutations affecting genes involved in Ca^2+^ and phosphatidylinositol signalling and their maternal component of expression, which complicates functional studies in the early embryo. However, amino acid sequence motifs in the Dof adaptor imply that signal transmission through these conserved signal transduction pathways does exist. The primary sequence of Dof shares similar domains with two vertebrate proteins, BCAP and BANK [55]. These proteins regulate B-cell receptor specific PI3K activation and calcium mobilization, respectively [61,62]. Dof also contains potential binding sites for PI3K, but its capability to bind PI3K and the role of PI3K in FGF signalling has yet to be confirmed. Dof protein possesses multiple clusters of functionally important tyrosine residues that provide docking sites for critical factors (Figure 3). Three potential binding partners, Csw/Shp2, Grb2/Drk and Src64B have been proposed to contribute to MAPK activation [50]. The protein sequence of Dof contains four consensus binding sites for Grb2/Drk [50], which recruits the guanine nucleotide exchange factor, Son of sevenless (Sos) to activate the small GTPase Ras85D [63] (Figure 2). GTP-Ras85D propagates the signal to the MAPK cascade via binding and activation of Draf (*pole hole*, MAPKKK) [64]. The Draf protein kinase elicits a phosphorylation cascade through successive phosphorylation of Dsor (Downstream of raf1, MAPKK) and ERK (*rolled*, MAPK) resulting in the phosphorylation of ETS (erythroblast transformation-specific) transcription factors [62]. The protein-tyrosine phosphatase Corkscrew (Csw), the *Drosophila* homolog of SHP2 also activates the Ras/MAPK pathway [56]. In mammalian cells Grb2 and SHP2 molecules signal together from FRS2α, suggesting that in the fly Csw and Drk may also form a complex with Dof allowing them to act in a concerted way. A direct link between Dof and Src64B was also shown, thus proposing a third route for FGFR-dependent MAPK activation [50] in which FGF signalling triggers the MAPK cascade via Src64B mediated activation of Draf [64]. The classical negative feedback loop of RTK signalling is mediated by Sprouty and dual specificity phosphatases. Although the biochemical mechanism of how Sprouty functions is not understood, Sprouty acts as an antagonist of FGF signalling [65]. Branchless induces the expression of Sprouty, which in turn inhibits FGF signalling and may be involved in fine tuning secondary branch sprouting in the cells closest to the FGF signalling centre. Whether Dof is involved in determining the specificity of FGF responses in the cell or merely acts as a modular scaffold protein is currently unclear. Recently, it was shown that cytosolic UDP-N-Acetylglucosamine (UDP-GlcNAc) levels are important for FGF signalling in *Drosophila* and that UDP-GlcNAc is required downstream of the receptor at the level of Dof [66]. Dof was shown to be *O*-GlcNAcylated in an *O*-GlcNAc Transferase dependent fashion indicating that posttranslational protein *O*-GlcNAcylation of Dof is required for its function in the pathway. The exact function of *O*-GlcNAcylation of Dof in FGF signal transduction remains to be determined.

Despite the many and potentially redundant mechanisms that activate the MAPK pathway downstream of FGFR binding, FGF signalling also clearly functions through MAPK-independent signalling routes (Figure 2). For example, activation of Ras is not sufficient to support migration in tracheal and mesodermal cells in *btl* and *htl* mutant embryos respectively [6,23,26,56]. These results implicate additional proteins downstream of the FGFR/Dof complex in transmitting the signal to regulators of the cytoskeleton and cell adhesion molecules [56]. Small GTPases of the Rho family are important regulators of actin cytoskeleton dynamics, and their activity is controlled by guanine nucleotide exchange factors (GEFs), GTPase activating proteins (GAPs) and guanosine nucleotide dissociation inhibitors (GDIs) [67]. The Rho-GEF Pebble (Pbl) is required for Htl-dependent contact formation between mesoderm and ectoderm cells during gastrulation and migration of mesoderm cells [6]. Pbl is an orthologue of the vertebrate proto-oncogene Ect2 and is essential for the actin-myosin contractile ring formation during cytokinesis [68]. Pbl/Ect2 facilitates GDP/GTP exchange on different members of the Rho family thus using distinct substrates according to the cellular context [68,69]. While Pbl activates Rho1 in cytokinesis, Pbl acts through a Rac-dependent pathway in FGF-dependent mesoderm morphogenesis [69]. Ect2 was also shown to activate Cdc42 in the regulation of cell polarity, cell shape and epithelial-mesenchymal transition (EMT) via the Par complex (Par-6, Bazooka/Par-3, aPKC and Cdc42) [70] yet evidence for Pbl using Cdc42 as a substrate is lacking [69]. The spatial distribution of Pbl in interphase cells indicates that the protein exerts its function at the cell cortex and structure function studies demonstrate that the Pleckstrin homology (PH) domain and a conserved carboxy-terminal tail of the protein are essential for its membrane association and activity [69,71]. How the specificity of Pbl towards distinct Rho GTPases is regulated and the role of the FGFR pathway in this process are important questions to be addressed in the future to help understanding how FGFR signalling impinges on the dynamics of the cytoskeleton during cell migration.

## 4. Cellular Responses Controlled by FGF Signalling

FGF signalling is involved in distinct developmental processes where it induces complex cellular behaviours. The nature of the given cellular response to an FGF stimulus depends on the endogenous and environmental signalling state of the cell. In *Drosophila* FGF signalling has particularly highlighted its functions in regulating cell migration, cell shape changes and EMT. A role of FGF signalling in cell proliferation has been demonstrated in the developing larval brain and the eye imaginal disc [30,31]. During CNS development, proliferation of cells of the perineural glia and cortex glia is both stimulated by Htl [31]. These subtypes of glia cells employ different molecular mechanism to regulate proliferation [31]. Cortex glia only requires Pyr provided by neurons while perineural glia cell number is controlled by Htl in parallel with the InR/TOR pathway [31]. In the eye imaginal disc, Pyr triggers glia proliferation prior to migration in an autocrine- or paracrine- fashion [30]. Despite this requirement in larval tissues, Htl and Btl appear both dispensable for cell proliferation during mesoderm and trachea morphogenesis in the embryo. Expression of a Btl-CA construct in the tracheal air sac primordium increased the number of tracheoblasts [72] and similarly Htl-CA caused expansion of the Eve-positive pericardial cells in the embryo [10,23]. However, this effect might be caused by a general over-activation of the Ras/MAPK pathway [26,73]. The role of FGF signalling for cell survival is less clear in *Drosophila*. During the growth of the tracheal ASP, EGFR, but not FGFR signalling, promotes cell survival and prevents apoptotic cell death in a MAPK-dependent fashion [35]. In another developmental context, FGF signalling is required for the survival of the migrating CVM cells [28]. In *htl* mutants these cells die prematurely and expression of the caspase inhibitor p35 rescues these cells and decreases cell death [28]. The pro-survival function of FGF might be secondary though, because *htl* mutant CVM cells detach from the TVM cells and lack of attachment to the extracellular matrix itself might induce cell death.

In many cases FGF signalling targets the transcription of genes that are important for initiating the differentiation programme of cells at discrete steps during development. In the tracheal system, cells express a particular set of transcription factors at each level of branch formation. Btl orchestrates primary branch formation, and it also activates the expression of genes controlling secondary branch formation, such as *pointed* and *sprouty*, and the terminal branch gene, *blistered* (*pruned*/DSRF terminal) [34,74]. During mesoderm differentiation *htl* is expressed in cardiac cells, somatic muscle precursors and visceral mesoderm cells [26]. Detailed analysis on *ths* and *pry* mutants as well as hypomorphic *htl* mutants has confirmed that FGF signalling contributes to the progressive restriction of mesoderm cell fates [15,25,26,73]. Hypomorphic *htl* alleles that exhibit no obvious defects in mesoderm spreading still show lack of Eve—expressing dorsal mesoderm cells indicating that Htl has dual functions [26]. Interestingly, Pyr, but not Ths is required for the specification of Eve-positive pericardial and dorsal muscle founder cells [15]. The expression of Eve and the differentiation of these dorsal mesoderm derivatives depend on several signalling inputs, including Wingless (Wg) and Decapentaplegic (Dpp) and it appears that Pyr represents a limiting factor in this signalling network [15,73]. Finally, specific body wall muscles depend on both FGF8-like ligands, Pyr and Ths, for their correct specification [15].

FGF signalling is responsible for inducing and controlling cell migration during *Drosophila* morphogenesis. Migrating cells have been shown to move towards the source of FGF, indicating that FGF ligands can provide instructive guidance cues to cells during directional cell migration in development [16,28,34]. FGFs organise directional cell movements by functioning as chemo-attractants that induce the formation of cellular extensions in the direction of migration in tracheal and mesoderm cells [16,72]. Thus FGF signalling is capable to trigger the formation of cellular protrusions, filopodia and lamellopodia, by regulating dynamic organisation of the actin cytoskeleton [16,72]. During mesoderm migration, movement into different directions requires distinct molecular cues: Ths and Pyr for radial and Pyr for dorsal protrusive activity [6,16,23]. The organization of cell protrusions is controlled by small GTPases of the Rho family: Rac is used for dorsal protrusions and Cdc42 is involved in formation of radial protrusions [16,69]. It is unclear how this specificity for different Rho family GTPases is controlled on the level of ligand-receptor interaction. Additional factors, such as cell adhesion and FGF ligand concentration, could modulate signalling and determine the choice of small GTPases in regulating actin-rich protrusion dynamics. FGFR/Dof-dependent activation and localisation of RhoGEFs represents a potential key determinant for remodelling of the actin cytoskeleton. Ample evidence in other systems implies PtdIns in directing protrusive activity in directional cell migration. A likely scenario would be that FGF signalling controls the localisation of Pbl by altering the PtdIns composition of the plasma membrane at the site of FGFR activation [69,75,76]. Pbl’s localisation to the cell cortex is necessary for inducing protrusive activity and its PH domain and conserved carboxy-terminal tail are essential for its localisation and activity [69,76]. Regulators of PtdInsP, such as the PtdIns(4)P-5-kinase Pi5k59B exhibit genetic interaction with *pbl*[76]. These data suggest that localisation of certain PtdIns might provide spatial control over Pbl activity, and indeed PIP2 has been shown to accumulate at ectoderm-mesoderm contacts [76]. However, the PH domain of Pbl appears to lack a clear binding preference for specific PtdIns *in vitro*[76]. It will be important to determine whether changes in the lipid composition of the plasma membrane are indeed triggered by FGFR signalling and what the downstream effectors of Rac and Cdc42 in this pathway are to understand how cytoskeletal dynamics and cell adhesive properties are integrated both temporally and spatially within the cell.

Btl triggers protrusion formation in tip cells of migrating trachea during primary branching and air sac morphogenesis [35,72,77]. Although, the requirement of small GTPases for tracheal development is well established, it is not known how their activity is regulated by FGF signalling. Molecular factors that are required for migration of air sac tip cells are Ras and pointed, however it is unlikely that these factors are directly involved in protrusive activity of cells [35]. One possibility is that pointed and/or other factors downstream of Ras/MAPK regulate the expression of genes required for migration. Cell migration is a complex process and although FGF signalling plays a major role in regulating dynamic cell protrusions, it could also act on processes other than protrusion formation. For example, FGF signalling might regulate adhesion and polarity of cells directly during complex processes like EMT.

EMT is defined as a series of coordinated changes in cell polarity, cell-cell adhesion, transcription, and motility [78]. The transcription factors Snail (Sna) and Twist (Twi) are key regulators of EMT. In the *Drosophila* presumptive mesoderm*,* Sna down-regulates epithelial genes, including E-cadherin and Crumbs while Twi induces mesoderm genes such as N-cadherin. While the cadherin switch represents a well-established milestone in EMT, in *Drosophila,* E-cadherin protein is maintained during gastrulation indicating that posttranslational mechanisms have to be inferred in the remodelling of apical/basal polarity and cell adhesion in presumptive mesoderm cells [16]. Redistribution of E-cadherin along the plasma membrane requires FGF signalling [16]. Pbl appears not required for loss of epithelial character, but it is essential for cell shape changes and gain of mesenchymal characteristics [6,75,76]. In the early mesoderm, mesenchymal characteristics can be monitored by protrusive activity and the formation of mesoderm/ectoderm contact which both depend on FGF signalling and Pbl, but not on MAPK activation [6,23]. These mesoderm-ectoderm cell-cell contacts are likely mediated by E-cadherin, which accumulates at the interface of the two germ layers in a Cdc42-dependent manner [16]. How Htl regulates E-cadherin localisation and the role that Cdc42 and potentially the PAR complex might play in this process remain important questions to be addressed. While integrin-mediated adhesion is not involved in directional mesoderm migration [16], FGF signalling is integrated with integrin-mediated adhesion and the extracellular matrix during CVM migration [79]. Thus FGF signalling does also interact with distinct regulators of the cytoskeleton and cell adhesion proteins in the cell. The dissection of the signalling network on which FGFR activation impinges remains a challenging but solvable problem in cell signalling research.

## 5. Conclusions

Over 20 years of FGF research in *Drosophila* have provided us with a profound knowledge of some of the important features of this pathway, from its molecular basis to their complex biological functions. The fly model has generated an immense toolbox, with which to address a number of key questions in the field. How is specificity for FGF/FGFR interactions within the cell generated and translated into distinct cellular outputs? How can FGFs provide spatial information and how is FGF ligand availability and concentration controlled in the extracellular space? And finally, how does the FGFR signal result in rapid changes in cell polarity, cell adhesion and cell shape during epithelial-mesenchymal transition? While classic genetic screens have identified many molecular players, novel mosaic screens in larval tissues, such as the air sac primordium, provide a unique opportunity to identify most, if not all, components that are required for FGF signal transduction in *Drosophila* with the prospect of translating this knowledge into mammalian organisms.

## References

[1] Glazer L., Shilo B.Z. (1991). The drosophila fgf-r homolog is expressed in the embryonic tracheal system and appears to be required for directed tracheal cell extension. Genes Dev.

[2] Itoh N., Ornitz D.M. (2010). Fibroblast growth factors: From molecular evolution to roles in development, metabolism and disease. J. Biochem.

[3] Zhang X., Ibrahimi O.A., Olsen S.K., Umemori H., Mohammadi M., Ornitz D.M. (2006). Receptor specificity of the fibroblast growth factor family. The complete mammalian fgf family. J. Biol. Chem.

[4] Klambt C., Glazer L., Shilo B.Z. (1992). Breathless, a drosophila fgf receptor homolog, is essential for migration of tracheal and specific midline glial cells. Genes Dev.

[5] Gryzik T., Muller H.A. (2004). Fgf8-like1 and fgf8-like2 encode putative ligands of the fgf receptor htl and are required for mesoderm migration in the *Drosophila gastrula*. Curr. Biol.

[6] Schumacher S., Gryzik T., Tannebaum S., Muller H.A. (2004). The rhogef pebble is required for cell shape changes during cell migration triggered by the *Drosophila* fgf receptor heartless. Development.

[7] Stathopoulos A., Tam B., Ronshaugen M., Frasch M., Levine M. (2004). Pyramus and thisbe: Fgf genes that pattern the mesoderm of *Drosophila* embryos. Genes Dev.

[8] Beiman M., Shilo B.Z., Volk T. (1996). Heartless, a *Drosophila* fgf receptor homolog, is essential for cell migration and establishment of several mesodermal lineages. Genes Dev.

[9] Reichman-Fried M., Shilo B.Z. (1995). Breathless, a *Drosophila* fgf receptor homolog, is required for the onset of tracheal cell migration and tracheole formation. Mech. Dev.

[10] Michelson A.M., Gisselbrecht S., Buff E., Skeath J.B. (1998). Heartbroken is a specific downstream mediator of fgf receptor signalling in *Drosophila*. Development.

[11] Lee T., Hacohen N., Krasnow M., Montell D.J. (1996). Regulated breathless receptor tyrosine kinase activity required to pattern cell migration and branching in the *Drosophila* tracheal system. Genes Dev.

[12] Metzger R.J., Krasnow M.A. (1999). Genetic control of branching morphogenesis. Science.

[13] Yang X., Dormann D., Munsterberg A.E., Weijer C.J. (2002). Cell movement patterns during gastrulation in the chick are controlled by positive and negative chemotaxis mediated by fgf4 and fgf8. Dev. Cell.

[14] Ciruna B., Rossant J. (2001). Fgf signaling regulates mesoderm cell fate specification and morphogenetic movement at the primitive streak. Dev. Cell.

[15] Klingseisen A., Clark I.B., Gryzik T., Muller H.A. (2009). Differential and overlapping functions of two closely related drosophila fgf8-like growth factors in mesoderm development. Development.

[16] Clark I.B., Muha V., Klingseisen A., Leptin M., Muller H.A. (2011). Fibroblast growth factor signalling controls successive cell behaviours during mesoderm layer formation in *Drosophila*. Development.

[17] Kadam S., McMahon A., Tzou P., Stathopoulos A. (2009). Fgf ligands in *Drosophila* have distinct activities required to support cell migration and differentiation. Development.

[18] Gisselbrecht S., Skeath J.B., Doe C.Q., Michelson A.M. (1996). Heartless encodes a fibroblast growth factor receptor (dfr1/dfgf-r2) involved in the directional migration of early mesodermal cells in the *Drosophila* embryo. Genes Dev.

[19] Winklbauer R., Muller H.A. (2011). Mesoderm layer formation in xenopus and *Drosophila* gastrulation. Phys. Biol.

[20] McMahon A., Reeves G.T., Supatto W., Stathopoulos A. (2010). Mesoderm migration in *Drosophila* is a multi-step process requiring fgf signaling and integrin activity. Development.

[21] McMahon A., Supatto W., Fraser S.E., Stathopoulos A. (2008). Dynamic analyses of *Drosophila* gastrulation provide insights into collective cell migration. Science.

[22] Murray M.J., Saint R. (2007). Photoactivatable gfp resolves *Drosophila* mesoderm migration behaviour. Development.

[23] Wilson R., Vogelsang E., Leptin M. (2005). Fgf signalling and the mechanism of mesoderm spreading in *Drosophila* embryos. Development.

[24] Shishido E., Higashijima S., Emori Y., Saigo K. (1993). Two fgf-receptor homologues of *Drosophila*: One is expressed in mesodermal primordium in early embryos. Development.

[25] Shishido E., Ono N., Kojima T., Saigo K. (1997). Requirements of dfr1/heartless, a mesoderm-specific drosophila fgf-receptor, for the formation of heart, visceral and somatic muscles, and ensheathing of longitudinal axon tracts in cns. Development.

[26] Michelson A.M., Gisselbrecht S., Zhou Y., Baek K.H., Buff E.M. (1998). Dual functions of the heartless fibroblast growth factor receptor in development of the *Drosophila* embryonic mesoderm. Dev. Genet.

[27] Dutta D., Shaw S., Maqbool T., Pandya H., Vijayraghavan K. (2005). *Drosophila* heartless acts with heartbroken/dof in muscle founder differentiation. PLoS Biol.

[28] Kadam S., Ghosh S., Stathopoulos A. (2012). Synchronous and symmetric migration of *Drosophila* caudal visceral mesoderm cells requires dual input by two fgf ligands. Development.

[29] Reim I., Hollfelder D., Ismat A., Frasch M. (2012). The fgf8-related signals pyramus and thisbe promote pathfinding, substrate adhesion, and survival of migrating longitudinal gut muscle founder cells. Dev. Biol.

[30] Franzdottir S.R., Engelen D., Yuva-Aydemir Y., Schmidt I., Aho A., Klambt C. (2009). Switch in fgf signalling initiates glial differentiation in the *Drosophila* eye. Nature.

[31] Avet-Rochex A., Kaul A.K., Gatt A.P., McNeill H., Bateman J.M. (2012). Concerted control of gliogenesis by inr/tor and fgf signalling in the *Drosophila* post-embryonic brain. Development.

[32] Ghabrial A., Luschnig S., Metzstein M.M., Krasnow M.A. (2003). Branching morphogenesis of the *Drosophila* tracheal system. Annu. Rev. Cell Dev. Biol.

[33] Jarecki J., Johnson E., Krasnow M.A. (1999). Oxygen regulation of airway branching in *Drosophila* is mediated by branchless fgf. Cell.

[34] Sutherland D., Samakovlis C., Krasnow M.A. (1996). Branchless encodes a *Drosophila* fgf homolog that controls tracheal cell migration and the pattern of branching. Cell.

[35] Cabernard C., Affolter M. (2005). Distinct roles for two receptor tyrosine kinases in epithelial branching morphogenesis in *Drosophila*. Dev. Cell.

[36] Chanut-Delalande H., Jung A.C., Lin L., Baer M.M., Bilstein A., Cabernard C., Leptin M., Affolter M. (2007). A genetic mosaic analysis with a repressible cell marker screen to identify genes involved in tracheal cell migration during *Drosophila* air sac morphogenesis. Genetics.

[37] Chanut-Delalande H., Jung A.C., Baer M.M., Lin L., Payre F., Affolter M. (2010). The hrs/stam complex acts as a positive and negative regulator of rtk signaling during *Drosophila* development. PLoS One.

[38] Ahmad S.M., Baker B.S. (2002). Sex-specific deployment of fgf signaling in *Drosophila* recruits mesodermal cells into the male genital imaginal disc. Cell.

[39] Murphy A.M., Lee T., Andrews C.M., Shilo B.Z., Montell D.J. (1995). The breathless fgf receptor homolog, a downstream target of *Drosophila* c/ebp in the developmental control of cell migration. Development.

[40] Sen A., Yokokura T., Kankel M.W., Dimlich D.N., Manent J., Sanyal S., Artavanis-Tsakonas S. (2011). Modeling spinal muscular atrophy in *Drosophila* links smn to fgf signaling. J. Cell Biol.

[41] Rapraeger A.C., Krufka A., Olwin B.B. (1991). Requirement of heparan sulfate for bfgf-mediated fibroblast growth and myoblast differentiation. Science.

[42] Nugent M.A., Edelman E.R. (1992). Kinetics of basic fibroblast growth factor binding to its receptor and heparan sulfate proteoglycan: A mechanism for cooperactivity. Biochemistry.

[43] Perrimon N., Lanjuin A., Arnold C., Noll E. (1996). Zygotic lethal mutations with maternal effect phenotypes in *Drosophila melanogaster*. II. Loci on the second and third chromosomes identified by p-element-induced mutations. Genetics.

[44] Hacker U., Lin X., Perrimon N. (1997). The *Drosophila* sugarless gene modulates wingless signaling and encodes an enzyme involved in polysaccharide biosynthesis. Development.

[45] Lin X., Buff E.M., Perrimon N., Michelson A.M. (1999). Heparan sulfate proteoglycans are essential for fgf receptor signaling during *Drosophila* embryonic development. Development.

[46] Kamimura K., Koyama T., Habuchi H., Ueda R., Masu M., Kimata K., Nakato H. (2006). Specific and flexible roles of heparan sulfate modifications in *Drosophila* fgf signaling. J. Cell Biol.

[47] Yan D., Lin X. (2007). *Drosophila* glypican dally-like acts in fgf-receiving cells to modulate fgf signaling during tracheal morphogenesis. Dev. Biol.

[48] Knox J., Moyer K., Yacoub N., Soldaat C., Komosa M., Vassilieva K., Wilk R., Hu J., de Vazquez Paz L.L., Syed Q. (2011). Syndecan contributes to heart cell specification and lumen formation during *Drosophila* cardiogenesis. Dev. Biol..

[49] Vincent S., Wilson R., Coelho C., Affolter M., Leptin M. (1998). The *Drosophila* protein dof is specifically required for fgf signaling. Mol. Cell.

[50] Csiszar A., Vogelsang E., Beug H., Leptin M. (2010). A novel conserved phosphotyrosine motif in the drosophila fibroblast growth factor signaling adaptor dof with a redundant role in signal transmission. Mol. Cell Biol.

[51] Itoh N., Ornitz D.M. (2004). Evolution of the fgf and fgfr gene families. Trends Genet.

[52] Tulin S., Stathopoulos A. (2010). Analysis of thisbe and pyramus functional domains reveals evidence for cleavage of *Drosophila* fgfs. BMC Dev. Biol.

[53] Klingseisen A (2010). Function of FGF8-Like1 and -2. *Drosophila mesoderm* Cell Migration. Ph.D. Dissertation.

[54] Wang Q., Uhlirova M., Bohmann D. (2010). Spatial restriction of fgf signaling by a matrix metalloprotease controls branching morphogenesis. Dev. Cell.

[55] Battersby A., Csiszar A., Leptin M., Wilson R. (2003). Isolation of proteins that interact with the signal transduction molecule dof and identification of a functional domain conserved between dof and vertebrate bcap. J. Mol. Biol.

[56] Petit V., Nussbaumer U., Dossenbach C., Affolter M. (2004). Downstream-of-fgfr is a fibroblast growth factor-specific scaffolding protein and recruits corkscrew upon receptor activation. Mol. Cell Biol.

[57] Wilson R., Battersby A., Csiszar A., Vogelsang E., Leptin M. (2004). A functional domain of dof that is required for fibroblast growth factor signaling. Mol. Cell Biol.

[58] Imam F., Sutherland D., Huang W., Krasnow M.A. (1999). Stumps, a *Drosophila* gene required for fibroblast growth factor (fgf)-directed migrations of tracheal and mesodermal cells. Genetics.

[59] Turner N., Grose R. (2010). Fibroblast growth factor signalling: From development to cancer. Nat. Rev. Cancer.

[60] Gabay L., Seger R., Shilo B.Z. (1997). Map kinase in situ activation atlas during *Drosophila* embryogenesis. Development.

[61] Okada T., Maeda A., Iwamatsu A., Gotoh K., Kurosaki T. (2000). Bcap: The tyrosine kinase substrate that connects b cell receptor to phosphoinositide 3-kinase activation. Immunity.

[62] Yokoyama K., Su Ih I.H., Tezuka T., Yasuda T., Mikoshiba K., Tarakhovsky A., Yamamoto T. (2002). Bank regulates bcr-induced calcium mobilization by promoting tyrosine phosphorylation of ip(3) receptor. EMBO J.

[63] Wassarman D.A., Therrien M., Rubin G.M. (1995). The ras signaling pathway in *Drosophila*. Curr. Opin. Genet. Dev.

[64] Xia F., Li J., Hickey G.W., Tsurumi A., Larson K., Guo D., Yan S.J., Silver-Morse L., Li W.X. (2008). Raf activation is regulated by tyrosine 510 phosphorylation in *Drosophila*. PLoS Biol.

[65] Hacohen N., Kramer S., Sutherland D., Hiromi Y., Krasnow M.A. (1998). Sprouty encodes a novel antagonist of fgf signaling that patterns apical branching of the *Drosophila* airways. Cell.

[66] Mariappa D., Sauert K., Marino K., Turnock D., Webster R., van Aalten D.M., Ferguson M.A., Muller H.A. (2011). Protein *O*-glcnacylation is required for fibroblast growth factor signaling in. Drosophila. Sci. Signal.

[67] Ridley A.J. (2006). Rho gtpases and actin dynamics in membrane protrusions and vesicle trafficking. Trends Cell Biol.

[68] Prokopenko S.N., Brumby A., O’Keefe L., Prior L., He Y., Saint R., Bellen H.J. (1999). A putative exchange factor for rho1 gtpase is required for initiation of cytokinesis in *Drosophila*. Genes. Dev.

[69] van Impel A., Schumacher S., Draga M., Herz H.M., Grosshans J., Muller H.A. (2009). Regulation of the rac gtpase pathway by the multifunctional rho gef pebble is essential for mesoderm migration in the *Drosophila gastrula*. Development.

[70] Liu X.F., Ishida H., Raziuddin R., Miki T. (2004). Nucleotide exchange factor ect2 interacts with the polarity protein complex par6/par3/protein kinase czeta (pkczeta) and regulates pkczeta activity. Mol. Cell Biol.

[71] Solski P.A., Wilder R.S., Rossman K.L., Sondek J., Cox A.D., Campbell S.L., Der C.J. (2004). Requirement for *C*-terminal sequences in regulation of ect2 guanine nucleotide exchange specificity and transformation. J. Biol. Chem.

[72] Sato M., Kornberg T.B. (2002). Fgf is an essential mitogen and chemoattractant for the air sacs of the *Drosophila* tracheal system. Dev. Cell.

[73] Carmena A., Gisselbrecht S., Harrison J., Jimenez F., Michelson A.M. (1998). Combinatorial signaling codes for the progressive determination of cell fates in the *Drosophila* embryonic mesoderm. Genes. Dev.

[74] Guillemin K., Groppe J., Ducker K., Treisman R., Hafen E., Affolter M., Krasnow M.A. (1996). The pruned gene encodes the *Drosophila* serum response factor and regulates cytoplasmic outgrowth during terminal branching of the tracheal system. Development.

[75] Smallhorn M., Murray M.J., Saint R. (2004). The epithelial-mesenchymal transition of the *Drosophila* mesoderm requires the rho gtp exchange factor pebble. Development.

[76] Murray M.J., Ng M.M., Fraval H., Tan J., Liu W., Smallhorn M., Brill J.A., Field S.J., Saint R. (2012). Regulation of *Drosophila* mesoderm migration by phosphoinositides and the ph domain of the rho gtp exchange factor pebble. Dev. Biol.

[77] Ribeiro C., Ebner A., Affolter M. (2002). *In vivo* imaging reveals different cellular functions for fgf and dpp signaling in tracheal branching morphogenesis. Dev. Cell.

[78] Lim J., Thiery J.P. (2012). Epithelial-mesenchymal transitions: Insights from development. Development.

[79] Urbano J.M., Dominguez-Gimenez P., Estrada B., Martin-Bermudo M.D. (2011). Ps integrins and laminins: Key regulators of cell migration during *Drosophila* embryogenesis. PLoS One.

